# How Does Emotion Influence Time Perception? A Review of Evidence Linking Emotional Motivation and Time Processing

**DOI:** 10.3389/fpsyg.2022.848154

**Published:** 2022-04-27

**Authors:** Philip A. Gable, Andrea L. Wilhelm, Bryan D. Poole

**Affiliations:** ^1^Department of Psychological and Brain Sciences, University of Delaware, Newark, DE, United States; ^2^Department of Behavioral and Social Sciences, Lee University, Cleveland, TN, United States

**Keywords:** emotion, time perception, motivation, affect, valence

## Abstract

Emotions have a strong influence on how we experience time passing. The body of research investigating the role of emotion on time perception has steadily increased in the past twenty years. Several affective mechanisms have been proposed to influence the passing of time. The current review focuses on how three dimensions of affect—valence, arousal, and motivation—are related to time perception. The valence-based model of time perception predicts that all positive affects hasten the perception of time and all negative affects slow the perception of time. Arousal is thought to intensify the effects of the influence of valence on time perception. In much of this past work, motivational direction has been confounded with valence, whereas motivational intensity has been confounded with arousal. Research investigating the role of motivation in time perception has found that approach-motivated positive and negative affects hasten the perception of time, but withdrawal-motivated affects slow the perception of time. Perceiving time passing quickly while experiencing approach-motivated states may provide significant advantages related to goal pursuit. In contrast, perceiving time passing slowly while experiencing withdrawal-motivated states may increase avoidance actions. Below, we review evidence supporting that approach motivation hastens the passing of time, whereas withdrawal motivation slows the passing of time. These results suggest that motivational direction, rather than affective valence and arousal, drive emotional changes in time perception.

## Introduction

Objective time and perceived time are distinct constructs. Perceived time is dependent on internal and external factors, such as affective states (James, 1890; Fraisse, 1978). Idioms and anecdotal evidence have corroborated decades of past research, which confirms that affective states permeate subjective experience (Izard, 2009) and change the perception of time. “Time flies when you are having fun.” “A watched pot never boils.” In this article, we first review research on how three dimensions of affect—valence, arousal, and motivation—are related to time perception. We then discuss recent research suggesting motivation, rather than valence or arousal, best explains the relationship between emotion and time perception.

## Affective Mechanisms Driving Changes in Time Perception

### Valence and Arousal

Valence is the subjective evaluation of an affective state that ranges from positive to negative (Lazarus, 1991; Harmon-Jones et al., 2011). Past research has shown that positively valenced stimuli are often judged for shorter durations than negatively valenced stimuli (Angrilli et al., 1997; Droit-Volet et al., 2004; Noulhiane et al., 2007). Conversely, negative stimuli are often judged for longer durations than positive or neutral stimuli (Stetson et al., 2007; Grommet et al., 2011).

Most of the studies comparing positive and negative valence, however, used highly arousing stimuli and failed to distinguish affective valence from arousal. Arousal is a “non-specific, energizing force that intensifies and strengthens either approach or withdrawal” (Bradley and Lang, 2007, p. 606); it can be measured subjectively (from “calm” to “excited”) or by activation of the sympathetic nervous system (Duffy, 1957, 1962; Gable and Harmon-Jones, 2013).

Highly arousing affective images are often judged as being displayed for longer durations than less arousing or neutral images (Gil and Droit-Volet, 2012). In addition, highly arousing faces are typically judged as being displayed for longer durations than neutral faces (Droit-Volet et al., 2004; Effron et al., 2006; Noulhiane et al., 2007; Tipples, 2008). Fayolle et al. (2015) induced arousal by giving participants a mild electric shock during a time bisection task. Participants judged stimulus durations as longer during trials that contained an electric shock than trials that did not. In another study (Mella et al., 2011), participants compared the duration of neutral, low-arousal negative, and high-arousal negative audio cues while skin conductance responses were recorded. When participants were asked to focus their attention on the emotional intensity of a stimulus, they reported the negative high-arousal cues as lasting longer and experienced higher autonomic arousal than when they focused attention on time. However, discrepancy between arousal and time perception led the researchers to conclude that there may not be a direct relationship between arousal and time perception.

In sum, the valence-based model of time perception predicts that all positive affects hasten the perception of time and all negative affects slow the perception of time. Arousal is thought to intensify the effects of the influence of valence on time perception. However, some of the research supporting a valence-based model relies on comparisons between positive and negative affective states. Such comparisons do not reveal whether positive states hasten perceptions of time, or whether negative states slow perceptions of time. Papers that compare neutral and affective stimuli do not test mechanisms driving the relationship between affect and time perception.

Much past research contradicts the valence model. For example, many studies have found that negative affects, like sadness and anger, can hasten the perception of time passing (Gil and Droit-Volet, 2009; Gable et al., 2016; Benau and Atchley, 2020; Yin et al., 2021). Thus, valence must not be the underlying mechanism causing affect to influence time perception. Perhaps another dimension of affect is altering the perception of time passing, one that is frequently confounded with affective valence. Below, we present evidence suggesting motivation as the affective mechanism influencing time perception.

### Motivation in Affect

The body of research on emotion and time perception has largely ignored the influence of motivation on time perception. Motivation refers to the action tendencies, or the urge to approach or withdraw, inherent in affect. Some affects are approach motivating, encouraging an organism to move toward a desired goal (Gable and Dreisbach, 2021). Conversely, some affects are withdrawal motivating, encouraging an organism to move away from an aversive stimulus (Gable and Harmon-Jones, 2013).

Approach and withdrawal motivation can be high or low in motivational intensity. For example, some positive affects are high in approach motivation (e.g., desire); other positive affects are low in approach motivation (e.g., contentment). Motivational intensity may also vary within affects of the same valence. Some negative affects are high in withdrawal motivation (e.g., disgust); other negative affects are low in withdrawal motivation (e.g., worry; Gable and Harmon-Jones, 2008, 2010b).

Motivational direction is not synonymous with valence. Much past work has confounded affective valence and motivational direction, such that all positive affects are assumed to be approach motivated and all negative affects are assumed to be withdrawal motivated. However, much research suggests this is not the case. For example, anger is a negative affect associated with approach motivation (Carver and Harmon-Jones, 2009; Harmon-Jones et al., 2011). Anger high in motivational intensity narrows an organism’s cognitive and attentional scope and can motivate goal acquisition (Gable et al., 2015; Threadgill and Gable, 2020). Similarly, sadness is a negative affect that can be associated with approach motivation (Higgins et al., 1997; Carver, 2004; Gable et al., 2016). Sadness low in motivational intensity broadens an organism’s cognitive and attentional scope in the face of lost goals (Gable and Harmon-Jones, 2010b) and can encourage an organism to seek new goals.

Arousal is often confounded with motivational intensity. In some cases, arousal can be a proxy for motivational intensity. Physiological and subjective measures of arousal can indicate the motivational intensity inherent in an affective state (Bradley and Lang, 2007). However, arousal and motivational intensity are not identical (Gable and Harmon-Jones, 2013). For example, arousal does not have a motivational direction. Highly arousing positive and negative affects are both high in arousal, but are opposite in motivational direction.

Much research has revealed the importance of motivational direction and intensity on cognitive and behavioral processes. We (Gable and Poole, 2012; Gable et al., 2016) proposed the mechanistic role of motivation in time perception, called the Motivational Dimensional Model of Time Perception. According to the model, approach motivation should hasten the perception of time and withdrawal motivation should slow the perception of time. Motivational intensity is predicted to enhance the influence of motivational direction. The body of research supporting the role of motivation has steadily increased. Below, we review evidence supporting that approach motivation hastens the passing of time, whereas withdrawal motivation slows the passing of time.

#### Approach-Motivated Positive and Negative Affects Hasten Time Perception

Past work based on a valence model of time perception confounded motivational direction with affective valence. We review work directly supporting the influence of approach motivation in hastening the perception of time in both positive and negative affects.

In one experiment, Gable and Poole (2012; Study 1) presented participants with pictures during a time bisection task. One third of pictures were neutral pictures, one third were positive pictures that elicited a low approach motivation, and one third were positive pictures that elicited a high approach motivation. Results revealed that participants perceived time passing faster after viewing the highly approach motivating pictures relative to the other picture types.

In a second experiment (Gable and Poole, 2012; Study 2), participants viewed the same set of pictures (dessert pictures), but approach motivation was increased in one group of participants, but not the other. To increase approach motivation, participants in one condition were told they would receive some of the desserts after the experiment, whereas participants in the other condition were not given these instructions. Afterward, participants reported how quickly time passed while viewing the pictures. Consistent with predictions, participants who expected to receive the dessert items experienced greater approach motivation and reported time passing faster than participants who did not expect to receive the dessert items.

Sadness and anger are negative affects associated with approach motivation. Recent research has found that sadness and anger may hasten the perception of time. In two experiments, Gable et al. (2016) tested whether sadness would shorten duration judgments. When viewing sad films or sad images, participants reported time passing faster than participants who viewed neutral films or images. Similarly, Benau and Atchley (2020) found that participants who recalled a sad memory showed bias toward shorter duration estimates in a temporal judgment task.

At first glance, research suggesting sadness hastens time perception appears to contradict other studies that link sadness and depression with a slowed perception of time, since both are often associated with withdrawal motivation (Thönes and Oberfeld, 2015; Kent et al., 2019). However, sadness can be either approach-motivating or withdrawal-motivating. For example, Gable et al. (2016; Study 4) had participants write about a past event while in a sad, approach-motivated state or while in a sad, withdrawal-motivated or inactive state. Consistent with predictions, participants who wrote about a sad event associated with approach motivation perceived time as passing more quickly during a subsequent retrospective temporal judgment task compared with participants who wrote about a sad event associated with withdrawal motivation or inaction.

Other work has examined the influence of anger on time perception. Some studies found that viewing static (Droit-Volet et al., 2004; Tipples, 2008; Kliegl et al., 2015) and dynamic (Li and Yuen, 2015) angry faces leads to a slowed perception of time or no effect at all (Grondín et al., 2015). Conversely, other studies have found that participants report time passing more quickly when viewing facial expressions depicting anger (Yin et al., 2021) and pain (Ballotta et al., 2018).

To test the role of approach motivation in negative affects more directly, Gable et al. (2016) directly tested whether manipulating approach motivation within negative states of sadness (Study 4) and anger (Study 5) would shorten the perception of time by directly manipulating approach motivation within the same affective state. States that were either high in approach motivation or low in approach motivation were evoked by having participants write about times when they experienced such states in the past. Results of both studies revealed that sad or angry states associated with approach motivation cause time to hasten relative to sad or angry states associated with inaction.

Together, these studies support that approach-motivated affective states hasten the perception of time. Importantly, approach-motivated affects appear to hasten the perception of time, regardless of whether they are positive or negative in valence. These results contradict the valence-based model of time perception and instead suggest motivation is responsible for changes in time perception.

#### Withdrawal-Motivated Negative Affect Slows Time Perception

Other recent work has investigated how withdrawal-motivated negative affects may be related to time perception. For example, Mioni et al. (2020) presented participants with disgust faces, happy faces, appetitive images (e.g., food, animals), and aversive images (e.g., infections, feces). Participants viewed images between 400–1600 ms and estimated how long each image was presented. When participants viewed disgust faces or aversive images, participants overestimated the passing of time. Similarly, Gable et al. (2016) presented participants with disgust images high or low in withdrawal motivation. Disgust images high in withdrawal intensity caused time to slow relative to disgust images low in withdrawal intensity and a neutral state. Together, these results suggest that negative affects high in withdrawal motivation slow the perception of time passing.

In another study, Matsuda et al. (2020) used a concealment manipulation to evoke guilt, a withdrawal-motivated negative affect. Participants were instructed to conceal an item from the experimenter during the study. Then, participants were made to feel guilty (vs. non-guilty) by viewing images of the concealed item (vs. another item). Individuals in the guilty condition had increased withdrawal motivation, perceived time as passing slower when viewing the images, and perceived all images as being displayed longer than participants in the non-guilty condition.

Other studies have manipulated withdrawal motivation by increasing physical effort, because effort is aversive (Richter et al., 2016). Hanson and Lee (2020) asked participants to run on an incline for 30 min at a pace that was somewhat hard or very hard. When participants were engaging in the hardest intensity of exercise, time slowed as compared to the easiest intensity. Similarly, Zhang et al. (2019) found that when individuals were asked to imagine lifting heavier items, they were more likely to overestimate time passing. These findings suggest that greater withdrawal motivation due to a highly effortful task can cause time to be perceived as passing more slowly.

## Discussion

Much recent research suggests that motivation may be a mechanism driving distortions of time perception in affective states. Approach motivation often hastens the perception of time for both positive and negative affects. Conversely, withdrawal motivation often slows the perception of time. Perceiving time passing quickly while experiencing positive affects may provide significant advantages related to goal pursuit. For example, affects high in approach motivation “should be associated with the perception of time passing faster…as organisms shut out irrelevant stimuli, perceptions, and cognitions” to accomplish a goal (Gable and Poole, 2012, p. 880). This is consistent with past research demonstrating a hastening of time when participants’ attention is distracted from processing temporal information (e.g., Macar et al., 1994). A hastened perception of time perception may encourage goal acquisition by increasing the hedonic value of objects or goals (Sackett et al., 2010) or helping an organism make predictions about the environment (e.g., perceive the presence of danger or goals; Meck, 2005). Conversely, affects high in withdrawal motivation may increase avoidance actions by increasing the perceived time spent in the presence of aversive objects or situations.

Research on neurotransmitters and neural structures further strengthens the link between affect, motivation, and time perception. For example, dopamine levels relate to reward processing and anticipation, and govern time estimation in mice (Soares et al., 2016). Further, dopamine levels in the dorsal striatum (Meck, 2006; Agostino and Cheng, 2016), the medial prefrontal cortex (Cheng et al., 2016), and the amygdala (Shionoya et al., 2013) reflect changes in time perception. Other neurotransmitters, such as norepinephrine, have also been implicated in attentional and temporal processing (e.g., Penney et al., 1996). These connections between time perception and neural structures and neurotransmitters related to motivation suggest there is a strong link between motivational processes and time perception.

### Attention in Time Perception

In the current review, we focus on the role of motivation, as opposed to affective valence, to influence time perception. However, some research on time perception posits that cognitive mechanisms of attention alter time perception during affective states (Angrilli et al., 1997). We discuss this model below.

Some past research has suggested that affective states may shift cognitive mechanisms of attention toward or away from key information (e.g., stimulus duration, bodily states, temporal processing), which may lead to a hastened or slowed time perception (Hawkins and Tedford, 1976). Burle and Casini (2001) found a hastening of time perception when attention was manipulated independently from arousal. In addition, Bar-Haim et al. (2010) found that briefly exposing anxious participants to threatening stimuli captured their attention but slowed time perception.

Much past work has linked attention with affective states (Easterbrook, 1959; Derryberry and Tucker, 1994; Gasper and Clore, 2002; Lacey et al., 2021). More recently, attentional scope has been linked with affective states high and low in motivational intensity. That is, positive and negative affects high in motivational intensity narrow attentional scope, whereas positive and negative affects low in motivational intensity broaden attentional scope (Gable and Harmon-Jones, 2008, [Bibr B23][Bibr B24]). Thus, it is possible that motivational intensity inherent in affect drives changes in participants’ attention, which, by extension, drives changes in time perception. However, positive and negative affects high in motivational intensity have demonstrated opposite effects on time perception (Gable and Poole, 2012, Experiment 3). Therefore, attention seems like an unlikely mechanism for the affective influence on time passing.

### Arousal in Time Perception

Past research has also suggested that arousal may drive changes in time perception, with highly arousing stimuli slowing the perception of time (Droit-Volet et al., 2004; Effron et al., 2006; Noulhiane et al., 2007; Tipples, 2008; Mella et al., 2011; Gil and Droit-Volet, 2012; Fayolle et al., 2015). Recent research has explored the connectedness of bodily mechanisms, such as being aware of one’s arousal state through heart rate (Di Lernia et al., 2018). However, results of this theory result in varied outcomes in regard to arousing stimuli and experience (Droit-Volet and Gil, 2016; Di Lernia et al., 2018). One reason for the inconsistent outcomes may be that past work has failed to operationalize arousal. Arousal might manifest as a subjective experience or a physiological response. This has led some researchers to conclude that arousal is “too broad of a concept to predict behavior, or indeed to convey meaning” (Neiss, 1990, p.101).

In addition, it is likely that past research has confounded arousal with motivation. For example, positive affects such as desire and negative affects such as anger are both high in arousal but are opposite in motivational direction (approach vs. withdrawal). Approach-motivated positive affects high in arousal hastened time perception relative to withdrawal-motivated negative affects high in arousal (Gable and Poole, 2012). In addition, directly manipulating approach motivation within two negative affects (i.e., sadness and anger) caused time to hasten (Gable et al., 2016), contradicting predictions of the arousal-based model of time perception.

However, because arousal is often used as a proxy for motivation (Bradley and Lang, 2007), research cannot completely rule out arousal as an explanation for changes in time perception. Future research should clarify what type of arousal is being measured or manipulated (i.e., subjective or physiological). Further, research should more closely examine whether motivation, but not arousal, *per se*, mediates the relationship between affect and time perception.

## Summary

The current review demonstrates that positive and negative affects varying in motivational direction have diverse effects on time perception. Both positive and negative affects related to approach motivation hasten time perception, whereas negative affects related to withdrawal motivation slow time perception. These results suggest that motivational direction, rather than affective valence and arousal, drive changes in time perception.

## Author Contributions

All authors listed have made a substantial, direct, and intellectual contribution to the work, and approved it for publication.

## Conflict of Interest

The authors declare that the research was conducted in the absence of any commercial or financial relationships that could be construed as a potential conflict of interest.

## Publisher’s Note

All claims expressed in this article are solely those of the authors and do not necessarily represent those of their affiliated organizations, or those of the publisher, the editors and the reviewers. Any product that may be evaluated in this article, or claim that may be made by its manufacturer, is not guaranteed or endorsed by the publisher.
